# Testosterone Therapy for Men With Age-Related Low Testosterone: Tempest in a Teacup

**DOI:** 10.1093/geroni/igab046.1815

**Published:** 2021-12-17

**Authors:** Shehzad Basaria

**Affiliations:** Harvard Medical School, Boston, Massachusetts, United States

## Abstract

Serum testosterone concentrations decrease in men with age, but benefits and risks of raising testosterone levels in older men remain controversial. In the T-Trials, a total of 790 men, age 65 and older, with a serum testosterone concentration of < 275 ng/dL and symptoms of sexual dysfunction, fatigue or physical dysfunction were randomized to either testosterone gel or placebo gel for 1 year. Treatment in the testosterone arm increased serum testosterone levels to the mid-normal range for young men. Testosterone replacement was associated with a significant increase in sexual activity (p < 0.001), libido and erectile function. In contrast, there was no improvement in vitality or physical function. Adverse findings included increases in non-calcified plaque formation and a higher rate of prostate events. In sum, testosterone treatment in older men was associated with modest benefits, while the risk on prostate and cardiovascular health remain unclear.

